# An integrated low vision service: Sri Lanka

**Published:** 2012

**Authors:** Sumrana Yasmin

**Affiliations:** Programmes Manager: South East Asia & Eastern Mediterranean, International Centre for Eye Care Education, Pakistan

**Figure:**
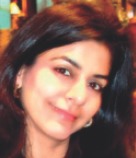
Sumrana Yasmin

Unusually for a low-or middle-income country, Sri Lanka provides free health services through government hospitals and other outlets.

Before, services for the estimated 140,000 people with low vision in Sri Lanka were provided by just three low vision clinics at tertiary hospitals; this meant that few people received the help they needed.

When a national eye care plan was developed in 2007, international non-governmental organisations (INGOs) such as Sightsavers and the International Centre for Eye Care Education were able to make a strong case for including low vision. As a result, and thanks to the support of the ministry of health, low vision was part of the national eye care plan from the outset. The necessary linkages with education, rehabilitation, and social services could also be established.

Implementation started in 2008 and the first priority was to strengthen the three clinics at tertiary level so they could provide visual skills training, orientation and mobility training, and counselling services for people with low vision.

Ten secondary level clinics, with strong referral links to the three tertiary clinics, were then established within existing district hospitals. These are easily accessible to most people, and offer comprehensive low vision assessment, prescription and dispensing of low vision devices, and training in the use of low vision devices. People with complicated needs are referred to the nearest tertiary low vision clinic for further management.

Ophthalmic technologists provide the services in the ten new secondary level clinics. These eye care practitioners were already working in the eye units at district level, with their salary paid for by government. Their availability, experience, and existing refraction skills made them the ideal group to train for this task.

Significant progress has already been made. By 2010, nearly 8,000 people (10% of which were children) had been helped, five times as many as in the previous three years.

The next step is to extend low vision services through community-based rehabilitation (CBR) services in order to reach under-served areas and groups; this will form part of existing CBR projects.

Programme planning and implementation is driven by the National Focal Person for Low Vision, Dr Saman Senanayake, who works in consultation with the ministry of health and INGOs. The expansion of the programme has been achieved through co-ordinated national planning, advocacy, human resource development, and the availability of affordable and low cost equipment.

